# Power and Fairness in a Generalized Ultimatum Game

**DOI:** 10.1371/journal.pone.0099039

**Published:** 2014-06-06

**Authors:** Giovanni Luca Ciampaglia, Sergi Lozano, Dirk Helbing

**Affiliations:** 1 Giovanni Luca Ciampaglia Center for Complex Networks and Systems Research, School of Informatics and Computing, Indiana University, Bloomington, Indiana, United States of America; 2 Sergi Lozano IPHES, Institut Català de Paleoecologia Humana i Evolució Social, Tarragona, Spain and Àrea de Prehistòria, Universitat Rovira i Virgili (URV), Tarragona, Spain; 3 Dirk Helbing Chair of Sociology, in particular of Modeling and Simulation, ETH Zürich, Zürich, Switzerland; University of Maribor, Slovenia

## Abstract

Power is the ability to influence others towards the attainment of specific goals, and it is a fundamental force that shapes behavior at all levels of human existence. Several theories on the nature of power in social life exist, especially in the context of social influence. Yet, in bargaining situations, surprisingly little is known about its role in shaping social preferences. Such preferences are considered to be the main explanation for observed behavior in a wide range of experimental settings. In this work, we set out to understand the role of bargaining power in the stylized environment of a Generalized Ultimatum Game (GUG). We modify the payoff structure of the standard Ultimatum Game (UG) to investigate three situations: two in which the power balance is either against the proposer or against the responder, and a balanced situation. We find that other-regarding preferences, as measured by the amount of money donated by participants, do not change with the amount of power, but power changes the offers and acceptance rates systematically. Notably, unusually high acceptance rates for lower offers were observed. This finding suggests that social preferences may be invariant to the balance of power and confirms that the role of power on human behavior deserves more attention.

## Introduction

At virtually all levels of human societies, socio-economic transactions are determined by the balance of power between parties. In fact, it is difficult to underestimate the importance of power in human social dynamics [1]. In the past, bargaining situations, and the Ultimatum Game (UG) in particular, have received large attention in the scientific literature, mostly because they highlight the importance of social preference in explaining human motivations beyond the standard rationality assumption [2]. In fact, under selfish rationality assumptions, as they have classicaly been made, Game Theory predicts that in the UG people would share as little money as possible and accept any share of money from others. However, since early pioneering work by Güth and colleagues [3], it has been observed experimentally that people have other-regarding preferences that favor fairness-oriented, pro-social outcomes. That is, small offers are rejected and accepted offers tend to come close to equity [2], [4]. This holds, albeit with certain caveats, across a large variety of cultural and social contexts [5], [6] (for a recent overview of the literature on ultimatum bargains, see the review by Güth and Kocher [7]).

Therefore, social preferences have been further incorporated into a fairness-oriented rational framework, which assumes inequality aversion [8]. Subsequently, various authors have developed evolutionary game theoretical models to explain human fairness preferences [9], [10]. Experimental results, however, indicate that some people have selfish preferences, while most people have fairness preferences, the degree of which varies from one person to another [11]. Such a broad spectrum of selfish and fairness preferences has been recently explained with a model that explains the evolution of both, a selfish “homo economicus” and an other-regarding “homo socials” [12]. In contrast to this model, which implies a level of cooperativeness and fairness that depends on the context, specifically on the behaviors in the social neighborhood, most models assume that preferences are stable with respect to the specific situation in which the bargain is performed.

Experiments show that situational and context-specific factors do affect preferences [13], and that what is perceived as selfish in one context can be construed as fair in another [14], [15]. Even unconscious cues may have a profound impact on the willingness of people to act fairly [16].

The effect of power on basic human cognition is clear: power is known to increase the amount of top-down stereotyping [17], to enhance goal-oriented behavior, and to increase the chances of goal-consistent action [18]. In general, the powerful tend to respond more to rewards, whereas the powerless more to threats [19], [20]. Power may act as an inhibitory factor of specific psychological systems [21] and has been associated with a decreased neural capability for processing social inputs [22].

The effects of power on social outcomes, however, are more nuanced: according to some authors, high social class, and hence higher power, is merely associated with more anti-social behavior [23]. However, others point out that exchange-oriented individuals (in the sense of the work of Clark and Mills [24]) when given power tend to act in a self-interested fashion, while in similar situations communally oriented individuals act more altruistically [25]. Power may also activate norms of social restraint in counter-intuitive ways [26], but so far previous scientific literature has devoted little attention to the role of power on individual preferences in bargaining situations, and it remains largely unclear.

In this work we set out to understand the role of bargaining power on social preferences, and we use the Ultimatum Game (UG) as a testbed for doing so. The UG is a stylized example of a bargaining situation in which the two players have different kinds of power. The proposer sets the stage and the responder has the power of the last word. We modify the payoff structure of the standard UG to investigate three situations: two cases in which the power balance is either against the proposer or the responder, and a balanced situation.

Our generalization of the classical UG is akin to manipulating power by means of a role-play with a well-defined balance of power. In the literature on the social psychology of power, it has been pointed out that having participants role-play a powerful position may trigger informal norms of social restraint [18], [26], [27]. To avoid possible interpretations of our results in terms of informal norms, we decided to design a completely double-blind setting in which confidentiality and anonymity were guaranteed among participants and between the participants and the experimenter.

We found that, while offers and acceptance rates vary almost predictably with the balance of power, other-regarding preferences, as measured by the amount of money donated by participants to the International Committee of the Red Cross (ICRC), do not change with the amount of power. We also find that, even when controlling for both fairness-oriented and self-oriented players, a significant fraction of bargains cannot be explained in purely rational terms.

This result shows that social preferences may be invariant to the balance of power. This is somewhat surprising, given the common belief that ‘power corrupts’ and in view of recent results about the increased chances of observing unethical behavior in higher strata of society [223]. It also adds to the growing literature on the role of power in affecting basic cognition [17]–[21], [25] and emphasizes that the role of power on human behavior deserves further scientific attention.

## Methods

### Manipulating power in the UG

In the experimental literature on the psychology of power, power is manipulated essentially in two ways: priming or role-playing [18]–[20]. In the first case, participants in an experiment are primed by asking them to recall a past experience in which they were either in a powerful or powerless situation. Despite the fact that priming only acts on the perception of power, several studies have shown that it is a powerful technique, eliciting measurable effects even at a neural level [22]. Moreover, depending on how the recall procedure is actually implemented (for example by having people write an essay) it may also let the experimenter assess its degree of activation in individual subjects – by measuring the valence of the essays in the case above. In our study priming was not a viable option because of the anonymity requirement; in writing about a past experience people might feel they are disclosing personal information and thus lose the perception of confidentiality with regards to the experimenter.

On the other hand power also can be manipulated by asking people to enact a situation in which some participants are actually assigned power over other participants. For example, in a manager/worker situation, Galinsky et al. [18] divided participants into teams and appointed a manager to each. The managers' task was to coordinate the work of the other team members, evaluate their results, and decide how much money they would be paid at the end of the experiment.

Because we were interested in studying the role of power in bargaining situations and how it may possibly affect other-regarding preferences, we chose the latter option, developing a version of the UG (described in greater detail in the following section) in which the payoff structure can assign more or less power to one of the two players.

Our experiment took place on the Web. Compared with a traditional, lab-based setting, the Internet offers several obvious advantages for implementing double-blind designs, since participants do not need to show up at the laboratory and are free to participate from home. People nowadays are more used to online collective interaction [28], and it also avoided the logistical problem of setting up multiple separate areas within our laboratory, which would have limited the number of participants that could simultaneously attend each session. Online experiments on the UG are becoming more common [29] thanks especially to online marketplaces such as the Mechanical Turk [30]. To our knowledge, what we report here is one of the earliest attempts to perform a completely double-blind design of the UG.

It is also worth noting that the confidentiality offered by the online setting provides a solution to another problem as well, raised by Galinksy et al. in the context of endowing participants with power instead of simply priming them with it [18], [26]: the activation of informal norms. In enacting situations such as the manager/worker one described above, informal norms of restraint could activate during the bargain and thereby influence part if not all of the observed behaviors. In other words, if exposed to a social context, the powerful might find it socially reproachable to exploit their bargaining power to make a selfish offer or to reject an equitable offer in favor of a more advantageous one. But because we could not interact with the participants, nor could they communicate with each other, this possibility can essentially be ruled out. Thus, any pro-social behavior we see in the experiment can be exclusively accounted to other-regarding preferences.

Our online setting also required another departure from the traditional way UG experiments are designed. In the classical UG, two people have to share a certain amount of money provided by the experimenter, which in the literature is known as “the cake”. It has been argued that this could lead participants to agree on an equal split just because the cake is a free gift from the experimenter [31]. To avoid this form of ‘windfall gains’, Berger et al. [31] required participants to wait for a certain amount of time before they could collect a constant payoff (the show-up pay) and asked them to decide how to split the waiting time.

Our experiment develops the setup of Berger et al. further [31]. There, sharing money was replaced by waiting time to avoid windfall gains, but despite this and also various efforts to increase the anonymity of the social interaction, the experiment delivered experimental results that are consistent with the original UG. Despite a number of further modifications in the Web experiments reported here, we find again similar sharing and acceptance behavior as in the classical UG, if the power between proposer and responder is balanced. However, varying the power of proposer and responder produces significantly different decision making results.

In our case, considering the online setup of the game, people could potentially spend their waiting time surfing on the web or taking a break from the computer, thereby defeating the whole concept of a bargain over time. We thus asked players to share the work of solving 300 additions of two single-digit numbers in case of acceptance and 600 in case of rejection. If the calculations were equally split, this amount corresponded to roughly 15–30 minutes of work for each participant. We chose simple arithmetic additions on the basis that the task should be simple enough for anyone and tedious enough to be perceived as an opportunity cost. Also, as the results of the sums were validated and stored on our server, this allowed us track effectively which participants completed the experiment without having to communicate with them.

### Ethics statement

The experiment was designed in compliance with the research ethics policies of the Decision Sciences Laboratory at ETH Zürich, and was approved by the ETH Ethics Committe (EK 2012-N-63). In particular, these policies prohibit any form of deception.

Participants were drawn from the student body of the Swiss Federal Institute of Technology (ETH) in Zürich. They were contacted via email (see File S1). For each experimental session 1,000 participants were randomly selected and contacted. Each participant was contacted only once. Participants were required to review a copy of the experimental policies before taking part in the experiment and agree to them, as a form of informed consent. They were not required to disclose any personal information in order to access the experiment website, and they were not assigned any data that could potentially allow us to track their actions individually during or after the experiment, such as cookies or personalized URLs. To access the website they were given a session password that was the same for all participants of any given session.

### Ultimatum Game with different power structures

Table 1 summarizes the payoff structure of our generalized UG. 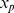 is the workload that the proposer offers to do, and 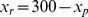 is the suggested share of the responder, if accepted (top row). Similarly, 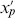 and 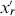 are the assignments in case of rejection. In our game offers can range from a minimum value of 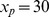 to a maximum of 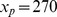. In the standard UG, if the second player rejects the offer of the first player, both players do not receive any money. In the variant of Berger et al., where players bargain over time, each of them was required to wait for the specified time [31]. In our case we decided to double the total workload (i.e. from 300 to 600 additions) and split it according to three different payoff functions reflecting the relative balance of power between players, corresponding to three different experimental treatments.

**Table 1:** Payoff functions for proposers and responders in our three experimental treatments.

Offer	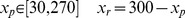		
Rejection	Weak proposer	Weak responder	Balanced
	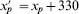	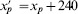	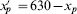
	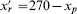	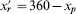	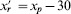

Top row: workloads 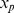 and 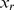 (number of two-digits additions) assigned, respectively, to the proposer and the responder in case of acceptance. Bottom row: workloads 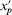 and 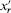 assigned in case of rejection in each of the experimental treatment.

We will call the three payoff functions taken into account “weak proposer”, “weak responder”, and “balanced situation”, as shown in Table 1 (bottom row). Their payoff structures are motivated by simple strategic considerations.

**Figure 1:**
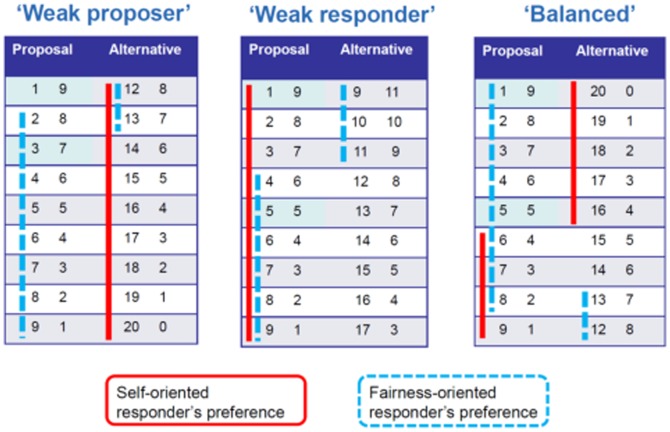
Rational choice predictions for self- and fairness-oriented preferences. For each panel, the proposal and the alternative workload in case of rejection are shown. The values displayed multiplied by 30 correspond to the actual number of arithmetic calculations to be done according to Table 1. Colors represent the orientation of responders in terms of their rational preferences (i.e. self- or fairness-oriented).

We consider two types of players: *fairness-oriented* and *self-oriented*. The payoffs were chosen such that the different treatments would be able to differentiate between self- and fairness-oriented preferences. Figure 1 graphically represents responders' expected preferences depending on their respective orientation (i.e. self- or fairness-oriented). From a self-oriented perspective, responders should always reject if this would reduce the number of calculations to be done. Thus, in a bargain involving self-oriented rational responders, they should reject all offers in the weak proposer and accept all offers in the weak responder treatment, while in the balanced treatment the response would depend on the concrete offer. Fairness-oriented responders would instead try to maximize fairness, i.e. minimize the difference in the number of calculations to be performed by both players.

Similarly, a self-oriented proposer should make the offer that minimizes the number of calculations to be done, and a fairness-oriented one should try to minimize differences in the number of calculations. However, in order to reach their goal, proposers have to make an assumption about the orientation of the responder. For example, take a self-oriented proposer in a “weak” responder situation. If the responder was also self-oriented, the optimal split of the workload would be 1–9, since it is the option assigning him the least workload among the ones potentially accepted by the responder (see red outlined cells in Fig. 1). However, if the responder was fairness-oriented, she should propose the split 4 - 6.

Notice that a wrong assumption about the responder's orientation might lead to a completely unsatisfactory outcome for the proposer [15]. For instance, in the example discussed above, if the proposer mistakenly assumes that the responder is self-oriented, the 1–9 split would be rejected in favor of a more fair 9–11 split.

**Figure 2:**
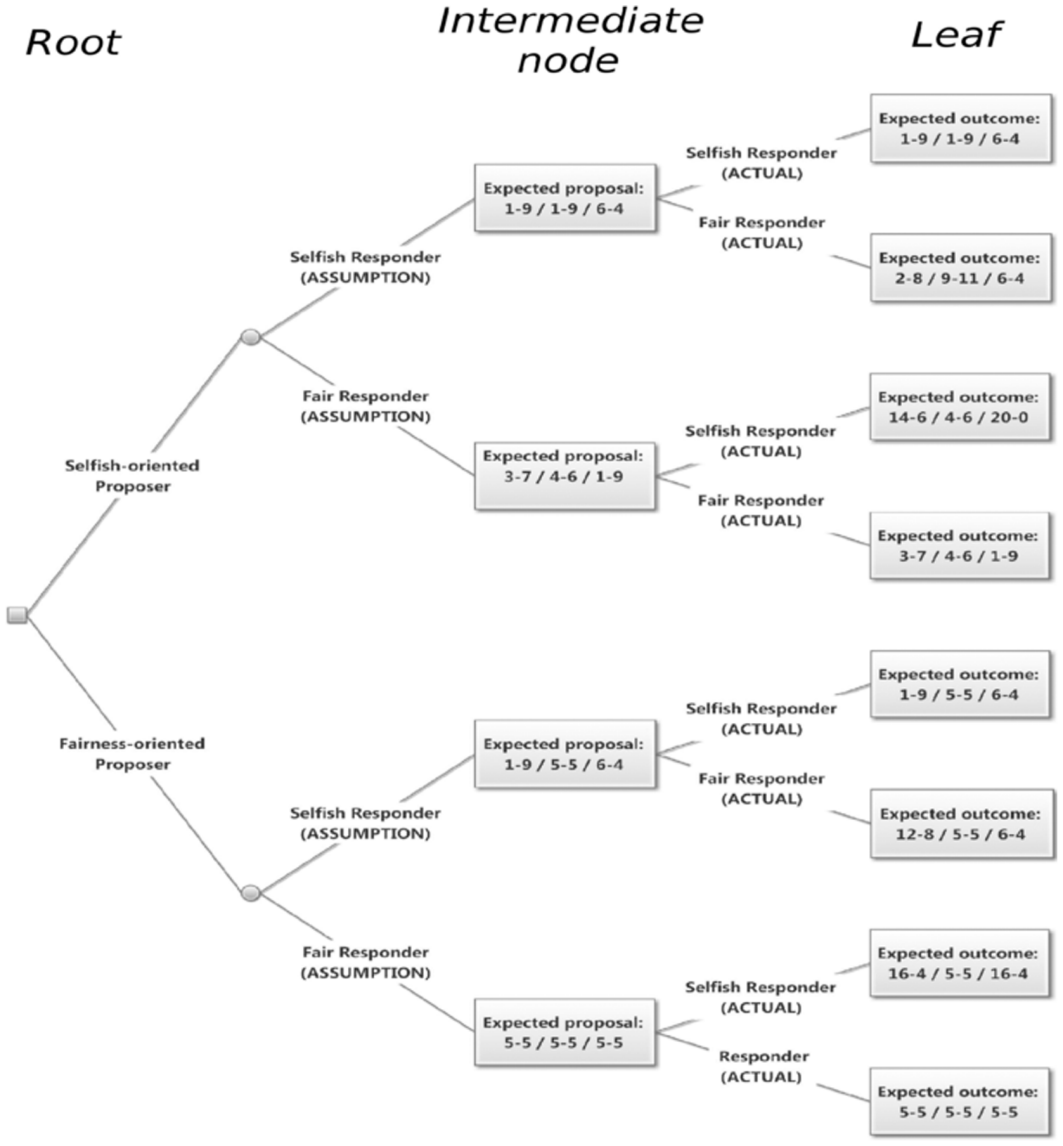
Rational choice predictions based on the proposer preference (root), proposer belief (intermediate nodes), and responder preference (leaves). In the intermediate nodes, the triplets indicate the expected proposal in each treatment (divided by 30). In the leaves, the expected outcomes (divided by 30) after the responder decision are shown.

Taking all this into account, we derived the expected behaviors of both players based on their respective preferences, i.e. assuming that a player is either self-oriented or fairness-oriented and the beliefs of the proposer concerning the preferences of the other player. Figure 2 shows the predictions of the model in form of a decision tree. Each level of branching represents the choice of one of the above variables. We start at the root node with the preference of the proposer; the intermediate nodes stand for the belief of the proposer regarding the responder's orientation (‘ASSUMPTION’), which determines the rational proposal. Finally, the branching leading to the leaves (‘ACTUAL’) represents the two possible beliefs of the responder and determines the expected outcome of the bargain.

Since the assumption of the proposer regarding the orientation of the responder might be wrong, our model captures a possible source of variability that is often observed in experimental settings [15].

## Results

### Turnout rate and game completion

We performed four experimental sessions, sending out 4,000 invitation emails in total. Weather conditions and time of the day were similar in all sessions. Altogether, 246 participants visited the website of the experiment. This amounts to a 6.15% show-up rate. Typical response rates in web surveys are slightly higher [32], but web experiments are usually also more demanding in terms of the involvement requested to participants, and ours was no exception.

Not all participants who logged into the website completed the experiment: 32 left before being matched to another player: 13 during the proposal phase (weak proposer: 7; weak responder: 3; and balanced: 3), and 5 after the game started (weak proposer: 2; balanced: 3). In summary, a total of 98 games in which both players stayed until the end of the experiment were completed. Broken down by treatment these are: 38 in weak proposer; 30 in weak responder; and 30 in the balanced treatment. Given the size of the workload they were required to bargain over (300 or 600 calculations), the number of participants who stayed until the end was surprisingly large.

### Effect of power on bargains

**Figure 3:**
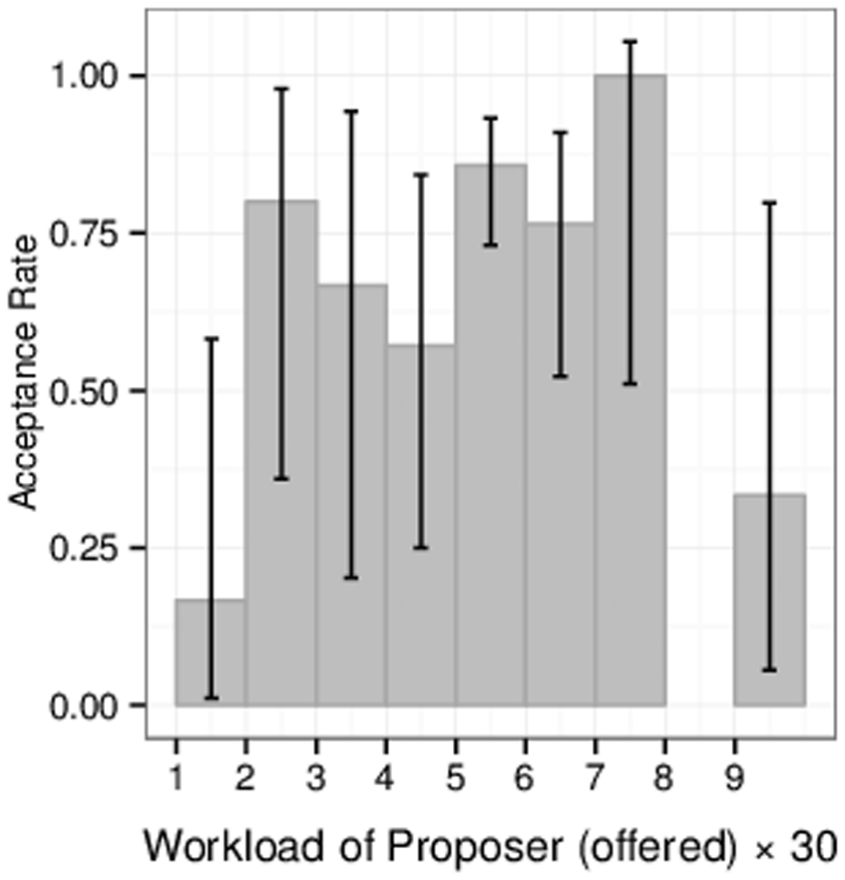
Acceptance rate as a function of proposed workload. Error bars are 95% Agresti-Coull approximate confidence intervals. We find monotonic acceptance rate, except for few hyperfair rejections.

Introduction of power led to strong divergence from previously reported observations in the UG. Figure 3 shows the proportion of accepted offers 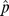 as a function of the workload 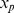 proposers offered to do. Notably, unusually high acceptance rates for lower offers (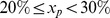) were observed (lower bound of the 95% Agresti-Coull approximate confidence interval 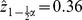). Here, all treatments are taken together. Moreover, there was a considerable amount of ‘hyperfair’ offers (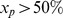), e.g. 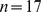 for 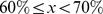. There were even 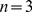 offers for 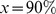 – the maximum allowed offer – and, looking at the responses to those offers, not all of them were accepted, which is very surprising.

**Figure 4:**
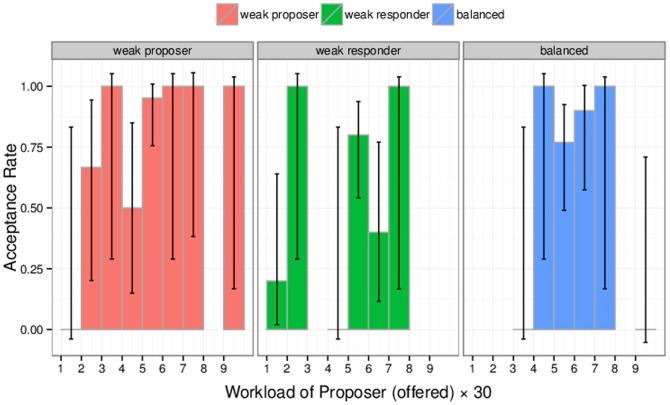
Probability of acceptance as a function of proposed workload and power treatment. Error bars are 95% Agresti-Coull approximate confidence intervals. We find monotonic acceptance rate, except for few hyperfair offers in the “balanced” treatment. Different treatment elicit different bargaining behaviors.

Compared with the traditional UG, varying the balance of power has thus dramatic consequences on the observed bargaining behavior. In particular, the somewhat surprising rejections for hyperfair offers (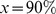) mentioned above have occurred in the “Balanced” situation, which could be explained as a form of inequality aversion (see Fig. 1). In line with our predictions, we found that different balances of power of the three treatments led to different sharing behaviors (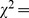
5.28, 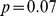). Figure 4 shows the breakdown by experimental treatment. In the balanced treatment (left panel), for example, the highest lower bound on the probability of acceptance does not correspond to the fair split but is attained for 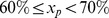. Moreover, almost no offer below 40% was made. In the other two treatments fair splits still correspond, in terms of lower bound, to the modal rate of acceptance, but, besides this detail, the distributions look very different from each other. In the weak responder case, the most advantageous offers for the proposers (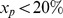) were made more often (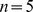 vs 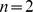), compared to the weak proposer case, and almost no offer (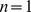 vs 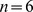) was observed in the range of moderately advantageous splits (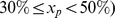) which is usually observed experimentally in the standard UG with no power.

These results are further substantiated by regression analysis of the proposed workload, using beta-distributed errors [33] and controlling for various confounding factors induced by the experimental design (see Section S3 of File S1); these include the donation, whether the show-up pay was eventually collected, the time spent by the proposer making the offer, the average outside temperature, which we use to measure opportunity costs, and others. The overall effect of power was significant (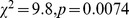). In particular, relative to a balanced scenario, proposers made smaller (i.e. more selfish), proposals in both the weak proposer (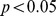) and weak responder (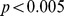) treatment. The log-likelihood of the model is 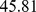 on 11 degrees of freedom, with a pseudo-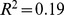. This quantity can be interpreted as the amount of variance explained by different treatments after controlling for relevant confounding factors.

### Donations and other-regarding preferences

Using a web form at the end of the game we asked participants whether they wanted to donate part of their show-up pay of 17 CHF to the International Committee of the Red Cross (ICRC). This method was used in order to measure other-regarding preferences without breaking the perceived level of confidentiality among participants. While the majority of subjects did not make any donations, 31.7% of them donated part of their money, with an average non-zero donation of 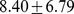 Swiss Francs. If non-donors are also taken into account, the average donation was instead 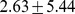 Swiss Francs.

To understand whether power affected other-regarding preferences, we matched each game according to the predictions of our model, see Figure 2. In particular we first determined the proposer's preference and their assumption about the responder's type by matching the observed offer 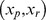 with any of the expected proposals. In doing so we used equally sized bins of 30 calculations. Then, based on the observed response, we determined the actual other-regarding preference of the responder.

The possible outcome of this exercise could result in matching a game to single, multiple, or no leaves of the decision tree at all, depending on the concrete case. This corresponds to assigning any of the three variables to either one, multiple, or no classes, respectively. For example, a weak responder game in which the proposal (divided by 30) is 5 - 5 and the response is acceptance is compatible with two different leaves (the fifth and the sixth from the top of Fig. 2), both related to a fairness-oriented proposer (lower branch, top level), who assumes she is dealing with a self-oriented responder (upper branch, ‘ASSUMPTION’). Regarding the preference of the responder, both branches (‘ACTUAL’), corresponding to either self- or fairness-oriented preferences would be compatible with the observed outcome, thus resulting in a ‘Multiple’ assignment.

**Figure 5:**
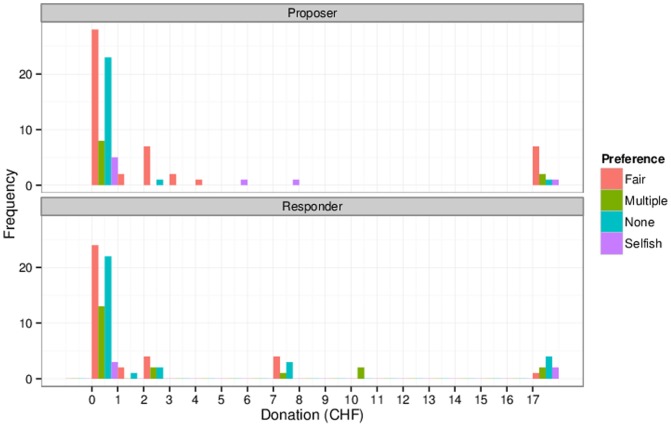
Donations by inferred orientation and role.

Applying this procedure on the experimental data resulted in 85 participants matching fairness-oriented preferences, 13 matching selfish-oriented preferences, 30 matching multiple classes, and 58 matching none of the classes. In particular, we found that 31.2% of the games were consistent with both players being fairness-oriented and a correct assumption of the proposer. On the other hand, 28% of the bargains did not match any of the predictions of the rational model (i.e., neither self- nor fairness-oriented). It is interesting to compare the donation data for both types of inferred other-regarding preferences. Figure 5 shows such a comparison, further broken down by the role (proposer or responder) of participants.

We then regressed the donated amount over the inferred preferences and the amount of power endowed to participants. We used the inferred preference instead of the actual proposed workload because what is a fair split depends on the treatment. Specifically, we considered a linear model including second-order interactions between the participants' role and their preference, the experimental treatment, the proposal, and the response. Moreover, as the data showed strong overdispersion (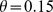), we employed a negative binomial regression model in lieu of a standard Poisson linear model. We tested several other models, including logistic regression, and only the negative binomial one could fit the data in a satisfactory manner.

The residual deviance of the model was 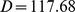 for 166 degrees of freedom, indicating a good fit (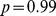). Table 2 reports the results of the regression analysis, including the coefficients and standard goodness-of-fit measures. Predictably, and consistent with the presence of other-regarding orientation, higher proposals tend to be associated with higher donations among proposers and, interestingly, with lower donations of responders. Proposers whose offers were rejected had a tendency to donate less, perhaps because the higher workloads resulting from rejection may have increased their sense of entitlement towards the earned pay. Crucially, however, in no treatment was power significantly related with increased or decreased donations. This is true for both roles, and supports the idea that other-regarding preferences may be invariant with respect to power. We also tested a simpler model with no interactions, but it could not capture any of the above results and was inferior to the model presented in Table 2. It is also worth noting that non-rational behavior, i.e. behavior consistent with neither self- nor fairness-oriented preferences (preference  =  ‘None’), was associated with significantly lower donations.

**Table 2:** Regression coefficients and goodness of fit.

	Neg. Bin. Model
Intercept	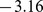
	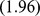
Role: Responder	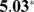
	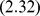
Preference: Multiple	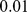
	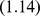
Preference: None	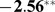
	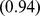
Preference: Selfish	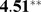
	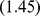
Weak proposer Treatment	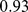
	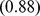
Weak responder Treatment	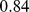
	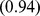
Proposal	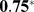
	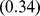
Response: Rejected	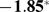
	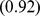
Role: Responder, Preference: Multiple	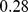
	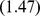
Role: Responder, Preference: None	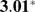
	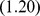
Role: Responder, Preference: Selfish	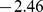
	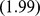
Role: Responder, Weak proposer Treatment	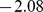
	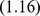
Role: Responder, Weak responder Treatment	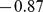
	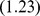
Role: Responder, Proposal	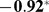
	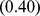
Role: Responder, Response: Rejected	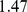
	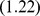
AIC	597.57
BIC	652.03
Log Likelihood	−281.78
Deviance	116.00
Num. obs.	182

Statistically significant coefficients are marked in bold.

***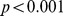, **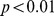, *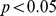.

### Summary and Discussion

Decades of experimental research have shown that in bargaining situations people do not follow the tenets of *Homo Economicus* and that other-regarding preferences, the hallmark of *Homo Socialis* [12], are the norm rather than the exception [6]. However, much is still to be understood about how pro-social behavior unfolds, and power is unarguably one of the most important factors affecting human interactions.

Here we studied how the balance of power in a bargaining situation affects other-regarding preferences of its actors by designing a double-blind variant of the classical one-shot UG to understand this. Our first finding is that, compared to a traditional setting, introducing power leads to strikingly different bargaining behaviors: whereas offers below 30% are seldom accepted in the classical UG, the Agresti-Coull 95% lower confidence bound on the estimated rate of acceptance for offers between 20% and 30% of the cake (all treatments) was 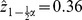, and manipulating the balance of power between players explained overall 19% of the variance in observed proposals. The fact that power shifted proposals towards smaller amounts of work thus provides further evidence that changing the process by which a bargain is performed changes also the perception of what is considered to be fair. Thus power may be affecting process-regarding preferences [14],[34].

While power changed the strategic component of the bargain dramatically, we also found that it did not affect the predisposition of people to donate to a humanitarian organization, i.e., other-regarding preferences do not seem to be influenced by assigning experimental subjects to a position of greater or smaller power. This is somewhat surprising, given recent results indicating that greater social status, and thus to a large extent greater power, is associated with greater chances of performing more selfish behavior [23].

Regarding the bargaining behavior of proposers, our anonymous setup allows us to rule out any explanation in terms of norms of social restraint. Fairness-oriented preferences show up in two independent ways – altruism and reciprocity. Regarding the latter, since we contacted our participants using their ETH student email address, we cannot entirely exclude the possibility that some form of group identification might have occurred. However, we have measured other-regarding behavior separately by asking people to donate to the ICRC — a generic universal institution. Therefore, we believe that explanations in terms of reciprocating behavior, at least for the donation behavior, can essentially be ruled out.

How can we make sense of this finding? We propose that the way participants framed the situation conditioned their propensity to engage in pro-social behavior or not. Power has been reported to enhance the likelihood of acting regardless of whether a social dilemma is framed as a public-goods or commons type of contribution [18], [25]. Thus, considering that donating to the ICRC corresponds to making a contribution (i.e. a public-good type of situation), this would predict more donations from the powerful. However, in order to avoid participants from inadvertently skipping the donation phase altogether, the donation screen was designed such that participants had to enter the amount of ‘0 CHF’ in case they did not want to donate any money. Consequently, they were prompted to act regardless of their intentions.

Given that they had to work in order to earn their reward, donating to the ICRC must have been framed as a loss of property that subjects were entitled to own. Further experimental work is thus needed to tease apart the effect of action, power, and loss aversion on other-regarding preferences. More work is also needed to incorporate these findings into a coherent theoretical framework. Recent work proposes an evolutionary framework for the emergence of pro-social and self-regarding preferences in a society [12], [29], [35]. It would, therefore, be interesting to assess whether such an approach can explain the invariance of preferences with respect to power or whether novel theoretical explanations need to be developed.

## Supporting Information

Figure S1 **Test screen.** Before taking part in the ultimatum bargain, participants were required to perform three calculations to get an idea of the subsequent workload.
(TIFF)

Figure S2 **Proposal screen.** This screen was showed to the proposer during the proposal phase.
(TIFF)

Figure S3 **Response screen.** This screen was showed to the responder during the response phase.
(TIFF)

Figure S4 **Structure of the experiment performed.** The online part of the experiment, during which subjects played the ultimatum game, is indicated by a solid line.
(TIFF)

File S1 **Supporting material for Power and Fairness in a Generalized Ultimatum Game.**
(PDF)

Table S1 **Control variables included in the analysis of the proposed workloads and responses.**
(PDF)
