# Supplementary figures and images for: Power and Fairness in a Generalized Ultimatum Game
Source: PLoS One. 2014 Jun 6;9(6):e99039. doi: 10.1371/journal.pone.0099039 (PMC4048244; doi:10.1371/journal.pone.0099039)

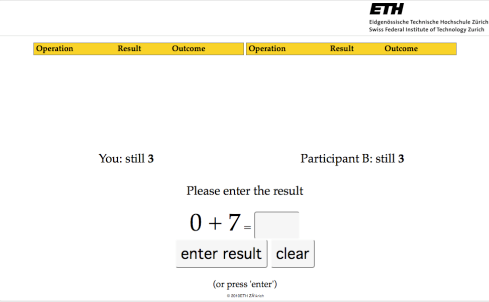

Supplement: Figure S1 — Test screen. Before taking part in the ultimatum bargain, participants were required to perform three calculations to get an idea of the subsequent workload. (TIFF) [file pone.0099039.s001.tiff]

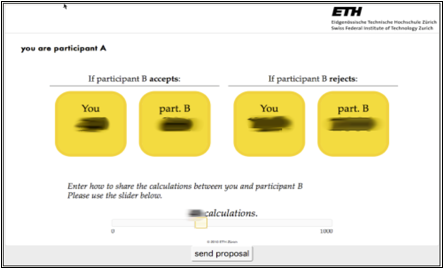

Supplement: Figure S2 — Proposal screen. This screen was showed to the proposer during the proposal phase. (TIFF) [file pone.0099039.s002.tiff]

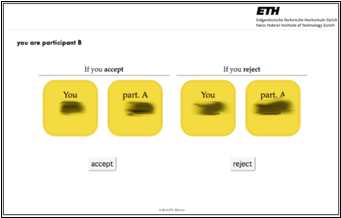

Supplement: Figure S3 — Response screen. This screen was showed to the responder during the response phase. (TIFF) [file pone.0099039.s003.tiff]

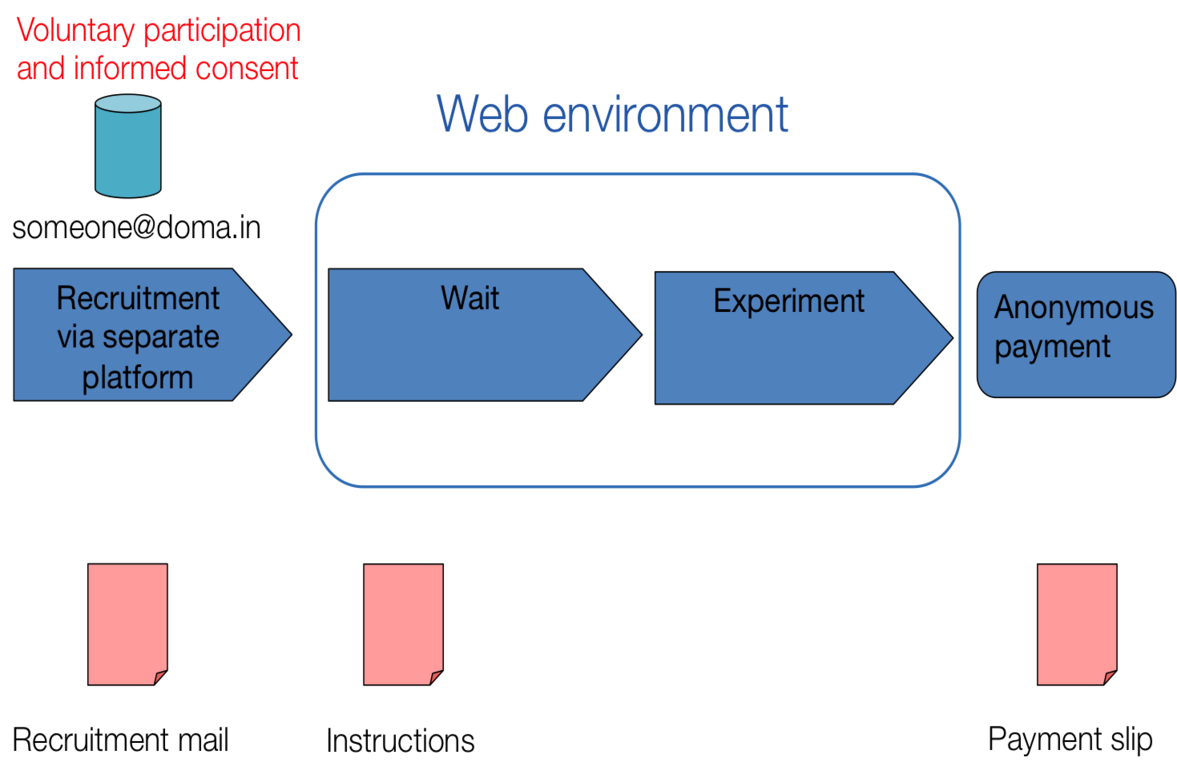

Supplement: Figure S4 — Structure of the experiment performed. The online part of the experiment, during which subjects played the ultimatum game, is indicated by a solid line. (TIFF) [file pone.0099039.s004.tiff]
