# Supplementary material for: Power and Fairness in a Generalized Ultimatum Game
Source: PLoS One. 2014 Jun 6;9(6):e99039. doi: 10.1371/journal.pone.0099039 (PMC4048244; doi:10.1371/journal.pone.0099039)
Supplement: File S1 — Supporting material for Power and Fairness in a Generalized Ultimatum Game. (PDF) [file pone.0099039.s005.pdf]

# Supporting material for *Power and Fairness in a Generalized Ultimatum Game*

## S1 Invitation e-mail

Potential participants were contacted with the following e-mail:

> Hello,
>
> we would like to invite you to take part in an online experiment. All you have to do is to login to http://www.hermes.ethz.ch/experiment today, Thursday 12, between 20:00 and 20:30 with this password: `seem-degree-rail-debt-wat`. You will be randomly matched with another participant and will play the game online. The experiment takes normally less than an hour. At the end of it you will receive a coupon worth 17.- CHF. You can exchange the coupon for cash at the official ETH cashier desk in the main building without providing your name or signature. Please do not talk with anyone about this experiment if you decide to join it. Thank you very much
>
> The Decision Science Laboratory (www.descil.ethz.ch)

All emails included a German translation of the above text.

## S2 Participant instructions

The following instructions were given to all participants before assigning them a role of the ultimatum game (i.e. proposer or responder).

**Introduction**

Dear participant,

You are currently taking part in a scientific experiment on sharing work (distributing tasks). **The experiment is estimated to take less than an hour, possibly much less**. Please make sure that you have enough time for this experiment. In order to receive the payment of 17 CHF, it is required that you finish the experiment according to these instructions. You will then receive a coupon with a code that can be exchanged for cash without disclosing your name or signature.

**The experiment**

This experiment involves two participants. We will call them **participant A** and **participant B** from now on. There are a number of simple tasks that have to be solved. Each of these tasks is a simple arithmetic calculation, like "2+2 = 4".

Participant A will make a proposal how to divide the work between both participants. Participant B will be asked to accept or reject the proposal of participant A.

In case of rejection, a higher number of tasks will be distributed. This distribution is coupled to and varies with the proposal of participant A. It will be shown to him/her when making the proposal but cannot be independently controlled.

**Starting the experiment**

After reading all pages of these instructions, please click on the START button below on this screen. If the other participant has not done so yet, you will have to wait for him or her to do so.

*IMPORTANT: It might take a several minutes for the other participant to log on and read the instructions, so please wait. The screen will refresh automatically. During this period, you can read a book, but please keep an eye on the screen. A sound will be played to notify you that the experiment has started.*

Next, you will be taken to this screen (see picture below), where you will have to solve 3 example tasks. This test will familiarize you with the tasks. After solving the 3 example tasks, you will be taken to the proposal phase.

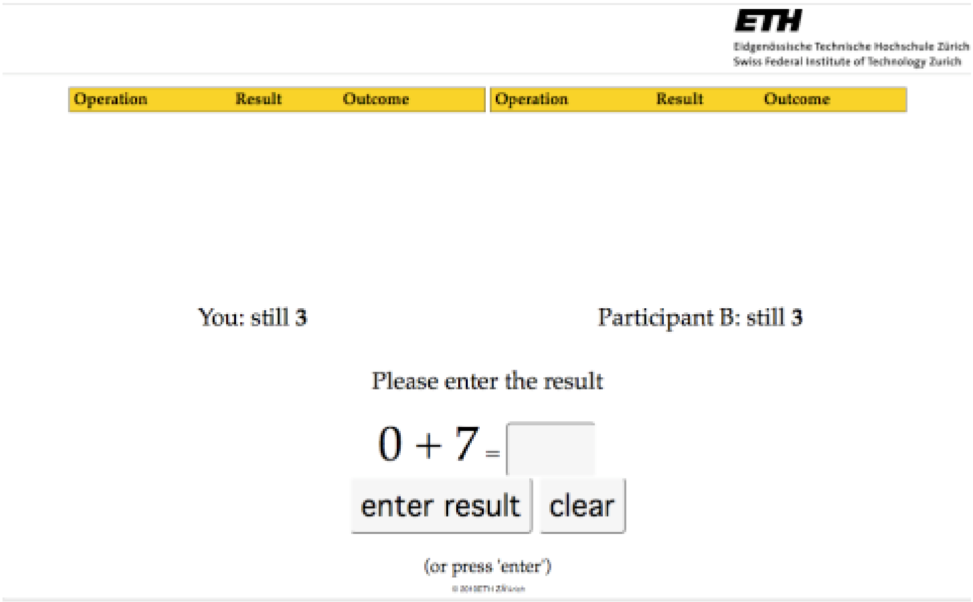


**Figure S1. Test screen.** Before taking part in the ultimatum bargain, participants were required to perform three calculations to get an idea of the subsequent workload.

### Proposal phase

If you are participant B, you will have to wait. The page will refresh automatically.

If you are participant A, you will see the screen below.

If you are participant A, you can use the slider in the middle of the screen to select how to share the work.[1]

The left panel lists your proposal. The right panel shows the amount of work that both of you will have to solve if participant B rejects the proposal.

[1] In order to avoid influencing the decisions of proposers, the control bar to determine the offer showed up only after the proposer clicked on the scale that represented the full parameter range.

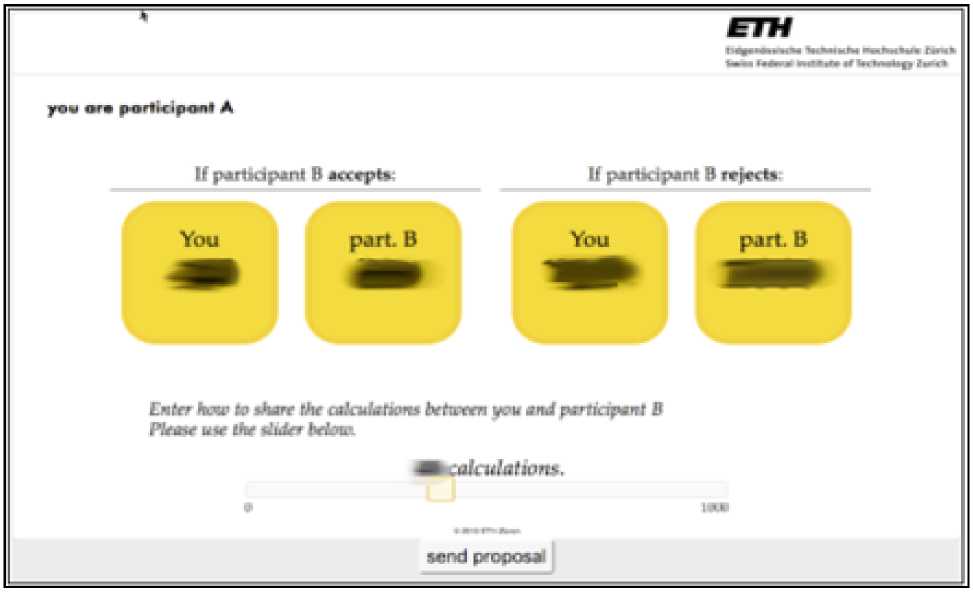


**Figure S2. Proposal screen.** This screen was showed to the proposer during the proposal phase. aptionProposal screen

To submit your choice to participant B, please click the button SEND PROPOSAL.

This will take you to the Reply phase.

## Reply phase

At this point, if you are participant A, you will have to wait. If you are participant B, this is the screen you will see:
Participant B must now click ACCEPT or REJECT to reply to the proposal of participant A. After this, the work phase will begin.

## Work phase

Both participants will be taken back to the calculation screen. If you are participant A, you will be notified of the decision of participant B. After that, both participants can start solving the calculations.

If a wrong value is inserted, you will be asked again to insert the correct value. Please note that you will receive the payment only if you solve all calculations that you have been assigned.

*Thank you for participating in this experiment!*

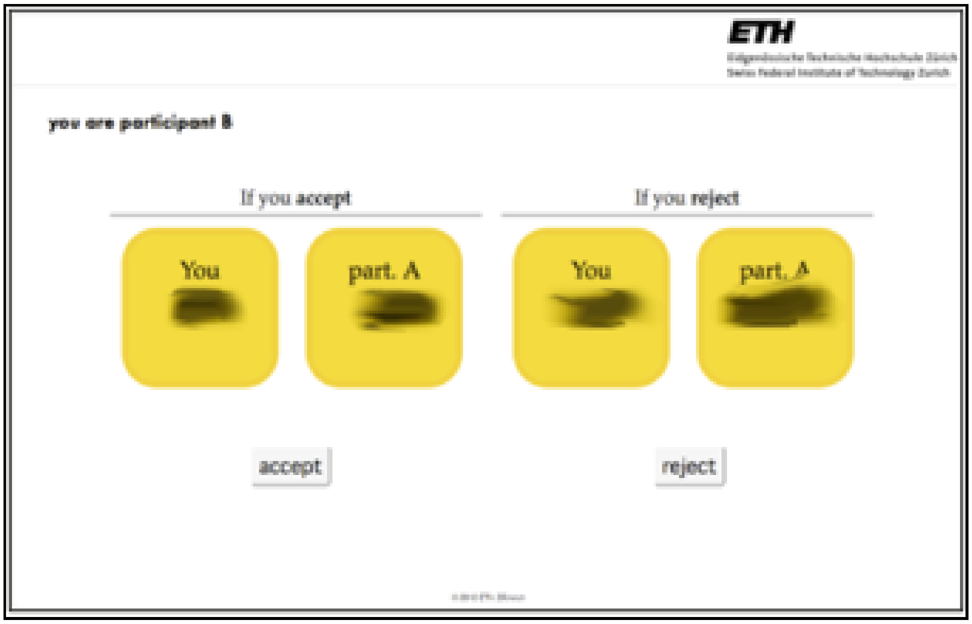


**Figure S3. Response screen.** This screen was showed to the responder during the response phase.

# S3 Experimental design

The ultimatum game itself was implemented as an interactive web application written with Django.[2] The application featured different phases in which participants, depending on their role, would either be requested to perform some action or wait for the input of the other participant. See Figure S4 for the sequence of steps of the online setup.

After signing in with the session password, participants were asked to read the instructions of the experiment (see Appendix for details) to confirm that they had fully read and understood them, and to wait to be matched with another person ('login wait', see Table S1). Participants were matched into (gaming) pairs following their order of arrival, thus ensuring that at every moment there was at most one participant waiting to be matched.

Because a long waiting time could influence the behavior of participants, we measured the duration of such time spans and controlled for it in the subsequent analysis. Additionally, we measured opportunity costs with the average outside temperature and the day of the week (all sessions occurred in the first part of the week). Table S1 summarizes all control variables used in the subsequent analysis. We introduce the other variables in the following.

Once matched, the server would randomly assign roles (proposer or responder) and the experimental treatment under which that specific instance of the ultimatum game would be performed (see Table **??**).

The two players were next asked to solve three test additions to get an idea of the workload and of the interactive interface. Once completed, the proposer was presented with the proposal screen, while the responder was asked to wait.

At this point in the game, proposers could choose how to share the workload by selecting with a slider the number of addition problems $x_p$ they intended to solve. For each choice of $x_p$, the interface would also show the associated value $x_r$ of addition problems assigned to the responder, as well as the values $\left(x'_p, x'_r\right)$ that the two would have to perform in case the responder rejected the offer. The rules for computing $x'_p$

[2] `http://www.djangoproject.com`.

**Table S1.** Control variables included in the analysis of proposed workloads and responses.

| Variable | Description |
|---|---|
| temp. (°C) | average outside temperature |
| day | day of week |
| login wait (min.) | time waited before matching occurred |
| treatment | factor: 'balanced', 'weak proposer', 'weak responder' |
| waiting time (min.) | *proposers*: time spent during proposal phase. *Responders*: time spent during proposal and response phase. |
| trials speed ($s^{-1}$) | average calculation speed |
| donation | fraction of reward donated to ICRC |
| payment received | was the reward collected after 30 days? |

and $x'_r$ in each treatment are shown in the bottom part of Table **??**. In order to avoid possible biases in the choice of $x_p$, the handle was not shown until the proposer clicked on the slider. Consistent with the literature on ultimatum games, in the following we normalized $x_p$ by the total amount of addition problems $x_p + x_r = 300$ and report results for the percentage share of workload proposed.

Next, the responder was asked to review the whole quadruple $\left(x_p, x_r, x'_p, x'_r\right)$ and to decide whether to accept or reject the offer. The proposer would then be notified of the decision of the responder and both were taken to the work screen where they had to solve all sums assigned to them.

Because we could not control how long subjects would take to formulate their choices, we recorded the time it took proposers and respectively responders to make their decisions and controlled for it in the subsequent analysis ('waiting time', see Table S1).

After entering correctly all sums in their workload, each player was taken to a final debriefing screen. At this point, to measure other-regarding preferences, players were asked whether they wanted to donate part of their monetary reward to the International Committee of the Red Cross (ICRC). Participants could choose not to donate any amount, in which case they had to insert an amount of 0 CHF. A printable voucher would then be generated, showing the amount to be paid, – i.e. 17 CHF minus the donation. Participants could redeem their voucher at the cash desk of the main building of ETH Zürich. In order to avoid counterfeiting, each voucher carried a unique, random code. At the cash desk, the cashier would insert the code in our database to check if genuine and mark the voucher as paid. This also enabled us to track, which participants collected their rewards and which did not. No signature or personal identifying information was required to cash the voucher.



git: b183286 on 2014-04-17

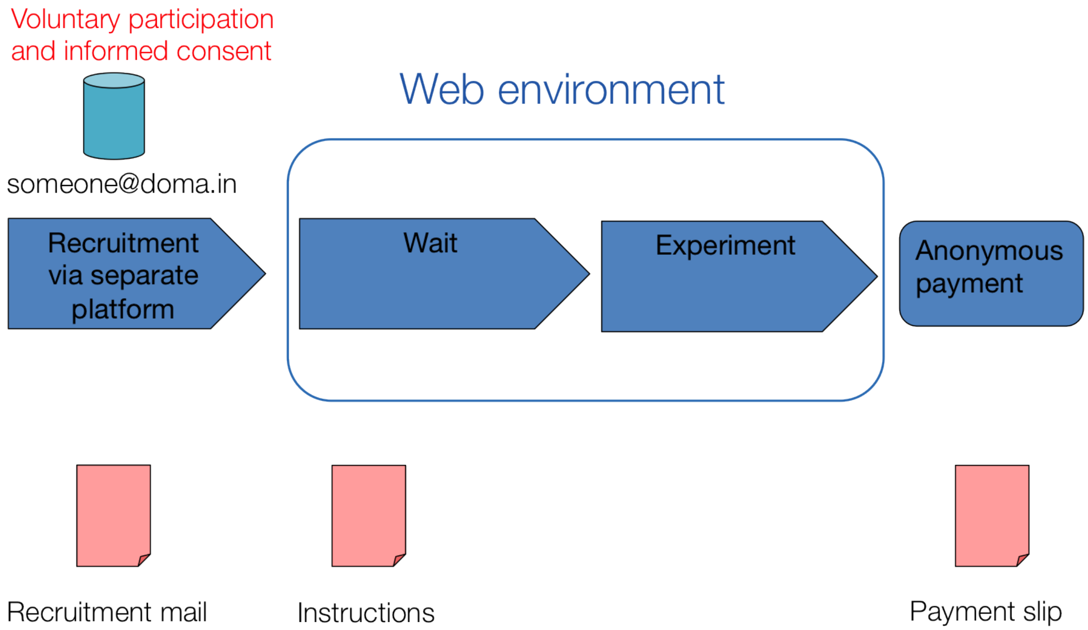


**Figure S4. Structure of the experiment performed.** The online part of the experiment, during which subjects played the ultimatum game, is indicated by a solid line.
