# Supplementary material for: Power and Fairness in a Generalized Ultimatum Game
Source: PLoS One. 2014 Jun 6;9(6):e99039. doi: 10.1371/journal.pone.0099039 (PMC4048244; doi:10.1371/journal.pone.0099039)
Supplement: Table S1 — Control variables included in the analysis of the proposed workloads and responses. (PDF) [file pone.0099039.s006.pdf]

Table S1. Control variables included in the analysis of proposed workloads and responses.

Variable	Description
temp. (°C)	average outside temperature
day	day of week
login wait (min.)	time waited before matching occurred
treatment	factor: ‘balanced’, ‘weak proposer’, ‘weak responder’
waiting time (min.)	*proposers*: time spent during proposal phase. *Responders*: time spent during proposal and response phase.
trials speed (s^{-1})	average calculation speed
donation	fraction of reward donated to ICRC
payment received	was the reward collected after 30 days?

git: b183286 on 2014-04-17
